# Complexities in Managing Wolf-Parkinson-White Syndrome: A Case of Congenital Inferior Vena Cava Anomaly With Azygos Continuation

**DOI:** 10.7759/cureus.69426

**Published:** 2024-09-14

**Authors:** Juan C Rivera-Martinez, Timothy Johnson, Safi Ahmed

**Affiliations:** 1 Internal Medicine, Lakeland Regional Health, Lakeland, USA; 2 Osteopathic Medicine, Nova Southeastern University Dr. Kiran C. Patel College of Osteopathic Medicine, Clearwater, USA; 3 Cardiology, Lakeland Regional Health, Lakeland, USA

**Keywords:** azygos vein, cardiovascular complications, catheter ablation, congenital venous anomalies, retrograde aortic approach, supraventricular arrhythmia, vc anomaly, wpw syndrome

## Abstract

The inferior vena cava (IVC) is a critical structure for venous return to the heart, and congenital anomalies of the IVC, though rare, can have significant clinical implications during procedures like catheter ablation for arrhythmias. In this case, a 26-year-old male presented with left-sided chest pressure after a routine exercise. Electrocardiography (ECG) revealed a delta wave and shortened PR interval, consistent with Wolf-Parkinson-White (WPW) syndrome, which involves an accessory electrical pathway leading to supraventricular tachycardia. Catheter ablation was planned to treat WPW syndrome; however, during the procedure, access to the right atrium via the IVC was obstructed due to an undiagnosed congenital IVC anomaly. Imaging revealed that the IVC was interrupted at the abdominal level, with venous return rerouted through an enlarged azygos vein into the superior vena cava. This anomaly prevented the use of the standard transvenous approach for ablation, and alternative approaches requiring specialized equipment and a highly skilled team available only at a tertiary care center were necessary. The patient was subsequently referred to such a center for further management. The IVC anomaly directly affected the procedural outcome, making pre-procedural imaging important when unexpected difficulties arise during ablation for WPW. This case demonstrates how congenital vascular anomalies may complicate standard electrophysiological procedures, especially in arrhythmia management. Future research should focus on developing alternative ablation techniques, such as the aortic approach, which may offer solutions for patients with vascular anomalies but require specialized facilities. While routine imaging for all WPW patients is not necessary, this case suggests that early imaging should be considered when procedural access is unexpectedly difficult. Establishing protocols and training for these complex scenarios is important for improving outcomes in similar cases.

## Introduction

The inferior vena cava (IVC) is the largest venous vessel below the thoracic cavity [1] and plays a critical role in returning blood to the heart. Although rare, congenital IVC anomalies can significantly affect cardiovascular health. These anomalies occur in approximately 0.3% of the healthy population and 0.6%-2% of individuals with cardiovascular disorders [2]. Specific defects, such as IVC aplasia and hypoplasia, affect between 0.005% and 1% of the population [3]. These anomalies not only alter the venous return, increasing the risk of deep vein thrombosis by 50-100 times, but may also be associated with a higher risk of arrhythmias [4]. The proposed mechanism for this increased risk is that abnormal venous return could alter the electrophysiological properties of the heart, predisposing patients to conditions like supraventricular arrhythmias, including Wolff-Parkinson-White (WPW) syndrome, which occurs in 0.9%-3% of the general population [5].

WPW syndrome is characterized by an accessory electrical pathway that can lead to supraventricular tachycardia [6]. Catheter ablation is the definitive treatment for WPW syndrome [7], requiring the navigation of a catheter through the femoral vein, up the IVC, and into the right heart [8]. However, congenital IVC anomalies may complicate this procedure by obstructing or altering the venous route, which can impede access to the right heart and prevent successful ablation.

## Case presentation

A 26-year-old male with no significant medical history presented with a two-week history of left-sided chest pressure localized to the left pectoral muscle following a routine of exercises. The pressure was exacerbated by left upper extremity movement but was non-exertional and non-radiating. The patient denied palpitations, tachycardia, shortness of breath, or any other associated symptoms, suggesting the discomfort was unrelated to his Wolf-Parkinson-White (WPW) syndrome and more likely due to physical activity. He also denied any history of smoking, alcohol, or recreational drug use, and there was no known family history of arrhythmias or congenital cardiac anomalies, indicating that the WPW and IVC anomaly were incidental findings in this patient.

**Figure 1:**
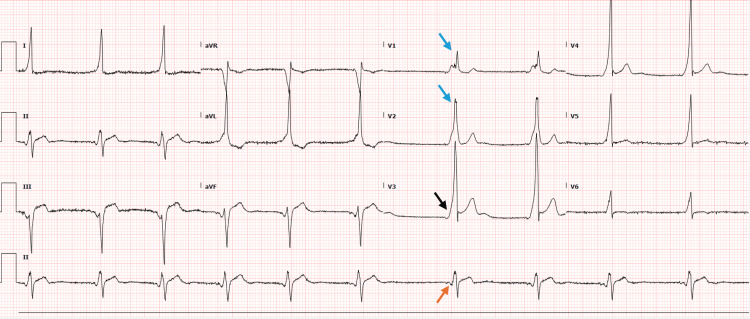
Electrocardiogram The blue arrows point at the RsR' pattern in lead V1 and V2, suggesting intraventricular conduction delay. The black arrow points to a delta wave, which represents early depolarization of the ventricles, contributing to the widening of the QRS complex. The orange arrow points at the shortening of the PR interval. These findings are consistent with Wolff-Parkinson-White syndrome.

**Figure 2:**
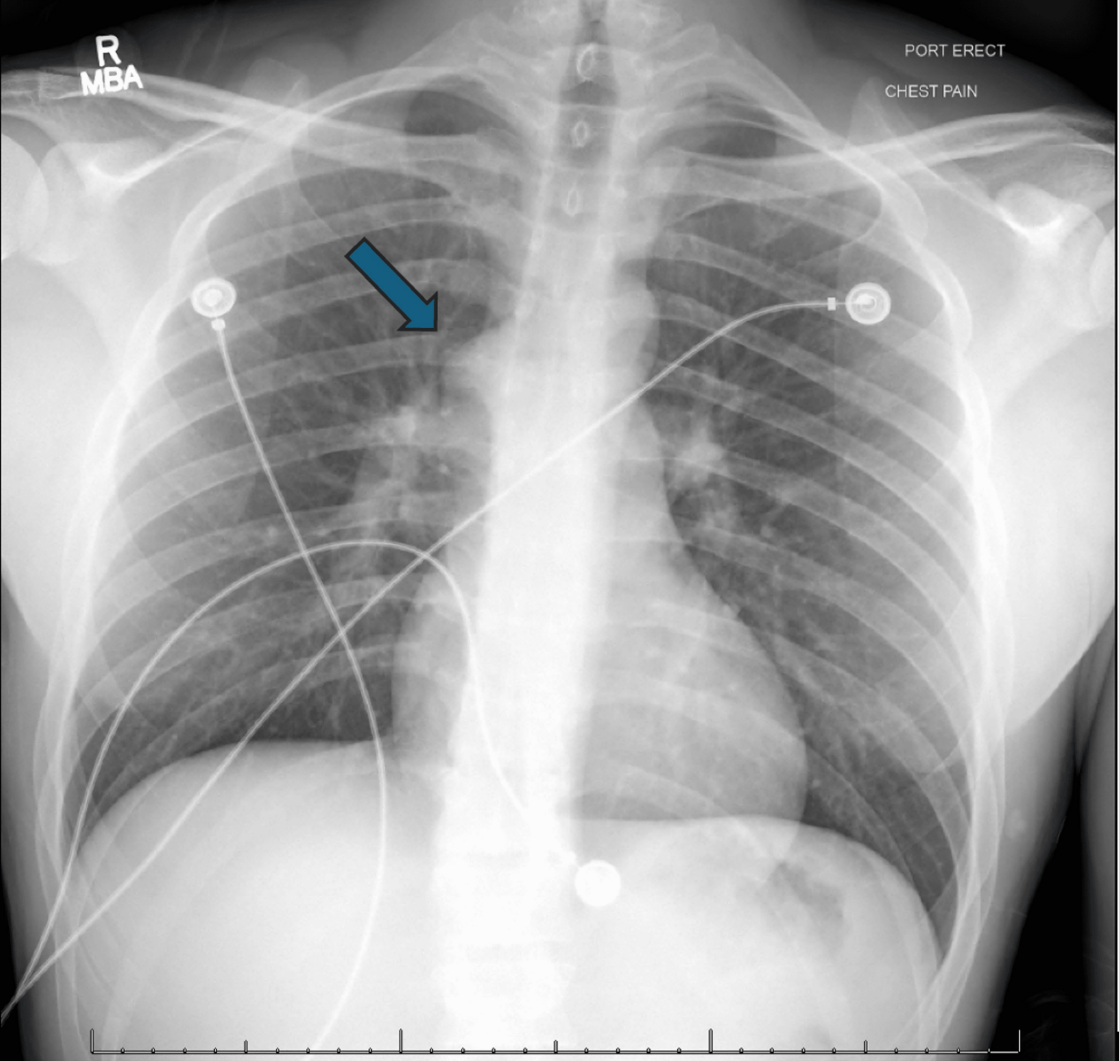
Chest x-ray. The blue arrow points at prominence in the azygous region. This prominence corresponds to the distended azygos vein, which was compensating for the interrupted IVC, serving as the primary route for venous return from the lower body to the superior vena cava.

On admission, routine laboratory tests were unremarkable. An electrocardiogram revealed a delta wave, shortened PR interval, widened QRS complex, and intraventricular conduction delay, diagnostic of WPW syndrome (Figure 1). A chest x-ray showed mild prominence in the azygos region (Figure 2), while an echocardiogram was normal.

**Figure 3:**
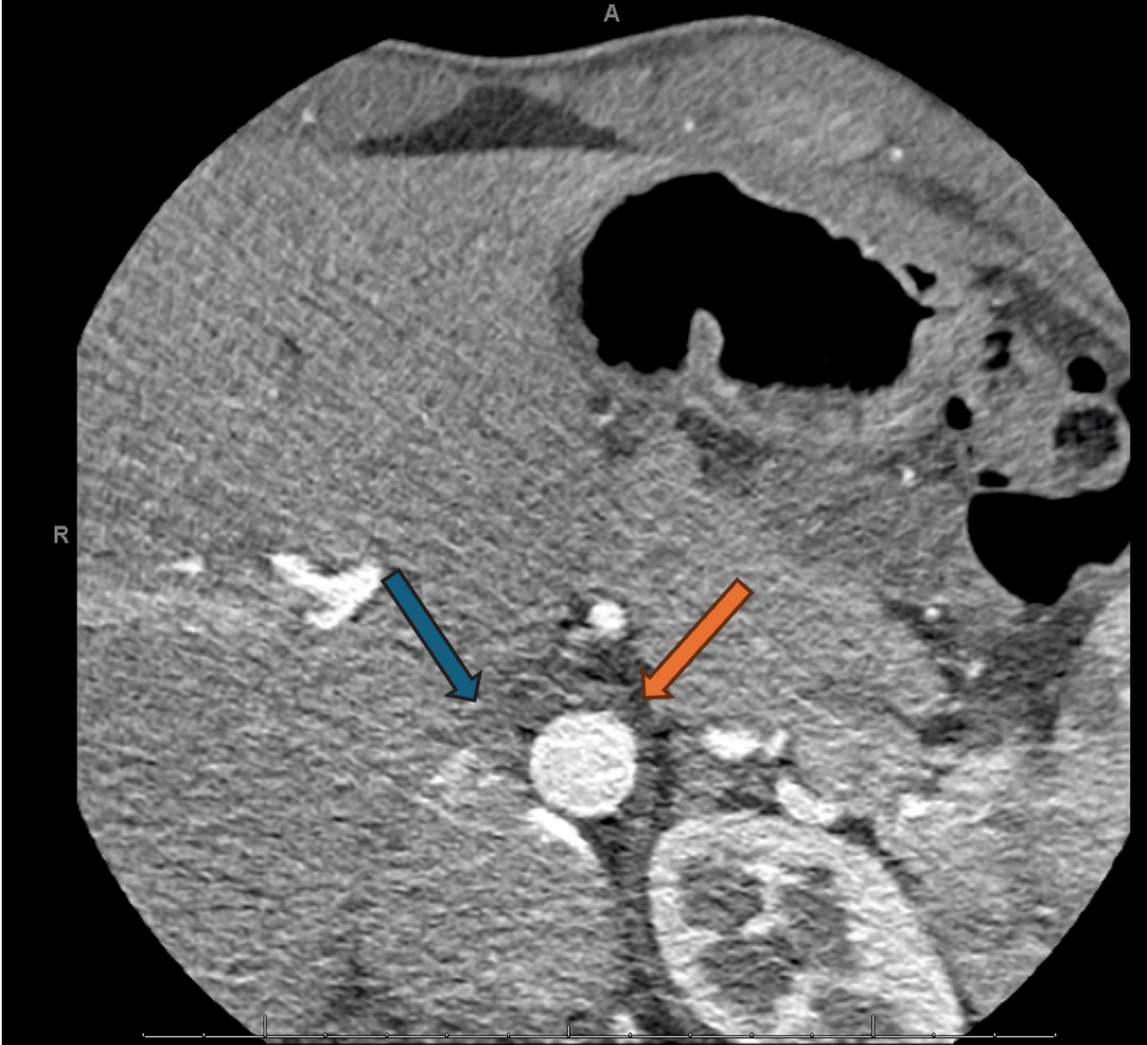
CT coronary angiogram: axial view at the abdomen level (liver), showing the aorta, and discontinuation of the inferior vena cava The blue arrow shows the discontinuation of the inferior vena cava at the abdominal level. The orange arrow shows the descending aorta for reference. These imaging findings were critical in guiding the decision to attempt a retrograde aortic approach for ablation, as the abnormal venous anatomy complicated standard access.

**Figure 4:**
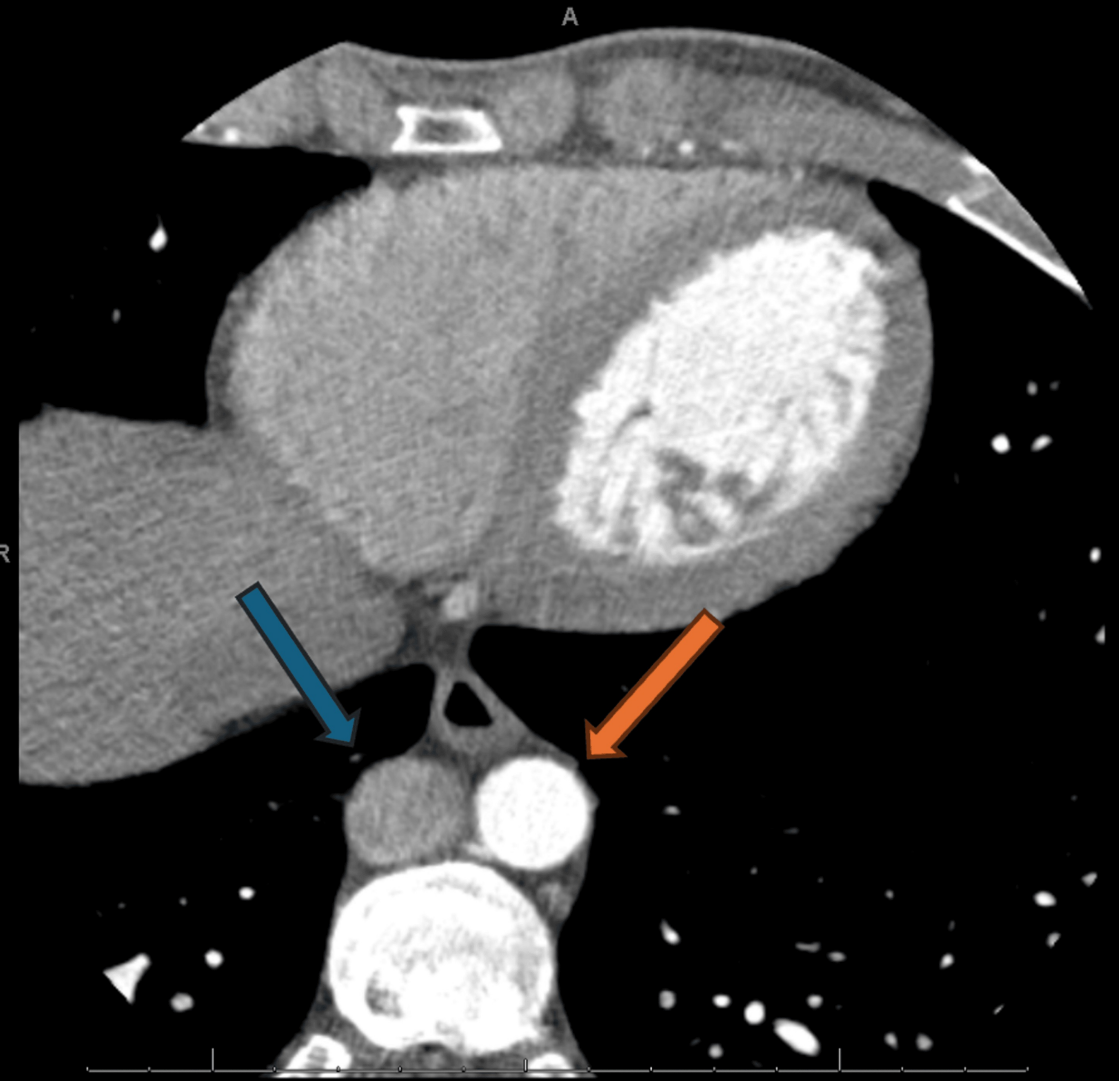
CT coronary angiogram: axial view at the thorax level, showing the continuation of the azygous vein The blue arrow shows the continuation of the azygous vein at the thoracic level. The orange arrow shows the descending aorta for reference. These imaging findings were critical in guiding the decision to attempt a retrograde aortic approach for ablation, as the abnormal venous anatomy complicated standard access.

**Figure 5:**
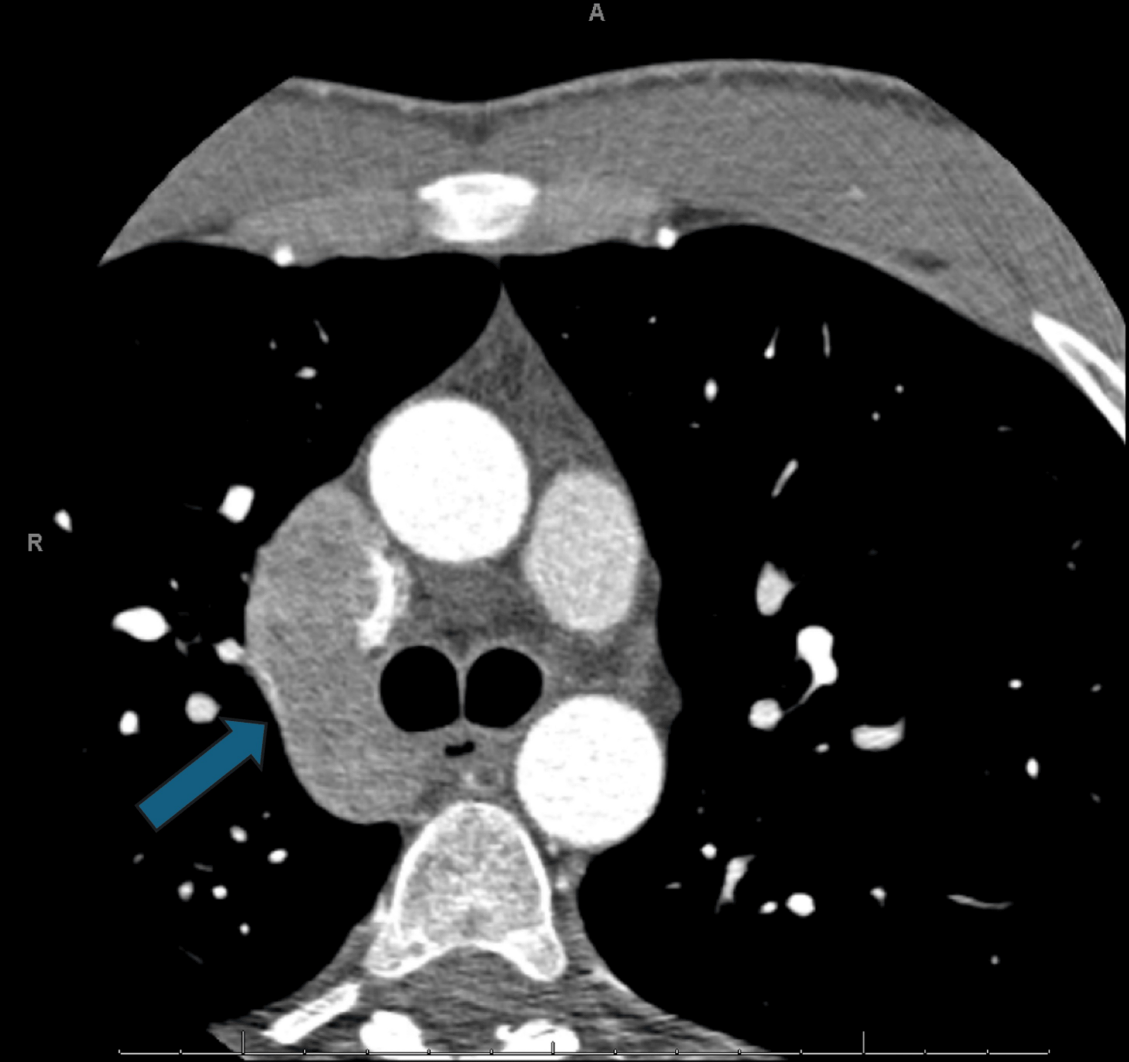
CT coronary angiogram: axial view at the thorax level, showing the distended azygous vein The blue arrow shows a dilated azygous vein, which ultimately connects to the superior vena cava. This distension suggests the azygos vein compensated for the interrupted IVC, carrying increased venous flow, which impacted the ability to advance the catheter into the right atrium via the transvenous route. These imaging findings were critical in guiding the decision to attempt a retrograde aortic approach for ablation, as the abnormal venous anatomy complicated standard access.

An electrophysiology study with catheter ablation was attempted to eliminate the accessory pathway. A quadripolar catheter was inserted via the femoral vein and inferior vena cava (IVC). However, resistance was encountered while attempting to access the right atrium. A fluoroscopic venogram was performed, which revealed contrast filling a structure presumed to be the azygos vein and superior vena cava, but no contrast filled the right atrium, indicating an obstruction in the IVC. Given the patient's chest discomfort, a computed tomography (CT) coronary angiogram with calcium scoring was performed to evaluate for acute coronary syndrome. This study incidentally noted an interruption of the IVC at the abdominal level (Figure 3), with continuation via the azygos vein at the thoracic level (Figure 4), and a markedly distended azygos vein draining into the superior vena cava (figure 5). 

Due to the IVC anomaly, the catheter could not be advanced into the right atrium, and the ablation could not be completed. Given the complexity of the case, the patient was referred to a tertiary care center where a retrograde aortic approach was considered an alternative method for ablation. The patient unfortunately was lost to follow-up.

## Discussion

This case presents a rare scenario of Wolf-Parkinson-White (WPW) syndrome complicated by congenital inferior vena cava (IVC) anomalies with azygos continuation, leading to significant diagnostic and therapeutic challenges. IVC anomalies often go undiagnosed until they complicate procedures like catheter ablation. In this case, the abnormal venous anatomy obstructed the transvenous route to the right atrium, requiring an alternative retrograde aortic approach.

Gayer et al. (2003) emphasized that IVC anomalies are frequently misinterpreted on imaging, potentially delaying diagnosis [2]. In our case, earlier recognition of the IVC anomaly could have aided pre-procedural planning. Similarly, Bass et al. (2000) highlighted the complications of azygos continuation in catheter-based procedures [9], which directly mirrors the challenges encountered here, where the distended azygos vein obstructed catheter access, necessitating a referral for a specialized ablation approach.

The relationship between IVC anomalies and arrhythmias remains underexplored, but abnormal venous return may alter atrial pressures, affecting electrophysiological properties. Only one similar case has been reported in the literature as far back as 1998 [10], underscoring the need for further research into the prevalence and management of IVC anomalies in arrhythmia patients.

Guidelines for referring patients to specialized centers or for advanced imaging remain limited. However, early referral should be considered when encountering unusual anatomy or procedural challenges. Imaging modalities such as CT venography or MR angiography can identify vascular anomalies early, allowing for alternative procedural planning. This can significantly improve outcomes in cases like ours.

Further studies should focus on understanding the prevalence of IVC anomalies in arrhythmia patients and developing alternative ablation techniques. Clinicians should incorporate advanced imaging in cases where standard procedures fail, ensuring early identification of anatomical anomalies and avoiding complications.

## Conclusions

This case demonstrates the significant challenges that can arise when managing WPW syndrome in the presence of a congenital IVC anomaly. The abnormal venous anatomy complicated the standard transvenous route for catheter ablation, requiring the use of a retrograde aortic approach. This situation shows the need for clinicians to be prepared for unexpected anatomical variations and the importance of specialized expertise in managing these cases. Early identification of vascular anomalies through advanced imaging, particularly in patients with arrhythmias, can guide procedural planning and prevent complications.

In complex cases like this, collaboration between cardiologists, radiologists, and vascular surgeons is crucial to providing comprehensive care. A coordinated approach ensures that patients with unusual anatomical challenges receive the most appropriate interventions. This case also points to the need for further research to explore the relationship between congenital IVC anomalies and arrhythmias, as well as to develop screening protocols that can detect such anomalies before procedures. Improving our understanding of these associations will lead to better treatment strategies and outcomes for patients with both conditions.

## References

[1] Shin DS, Sandstrom CK, Ingraham CR, Monroe EJ, Johnson GE (2019). The inferior vena cava: a pictorial review of embryology, anatomy, pathology, and interventions. Abdom Radiol (NY).

[2] Gayer G, Luboshitz J, Hertz M (2003). Congenital anomalies of the inferior vena cava revealed on CT in patients with deep vein thrombosis. AJR Am J Roentgenol.

[3] Kim H, Labropoulos N, Blake AM, Desai K (2022). Prevalence of inferior vena cava anomalies and their significance and impact in clinical practice. Eur J Vasc Endovasc Surg.

[4] Shafi I, Hassan AA, Akers KG, Bashir R, Alkhouli M, Weinberger JJ, Abidov A (2020). Clinical and procedural implications of congenital vena cava anomalies in adults: a systematic review. Int J Cardiol.

[5] Luca AC, Curpan AS, Miron I, Horhota EO, Iordache AC (2020). Paroxysmal supraventricular tachycardia in Wolff-Parkinson-White syndrome in a newborn - case report and mini-review. Medicina (Kaunas).

[6] Vătășescu RG, Paja CS, Șuș I, Cainap S, Moisa ȘM, Cinteză EE (2024). Wolf-Parkinson-White syndrome: diagnosis, risk assessment, and therapy - an update. Diagnostics (Basel).

[7] Niu G, Guo Y, Guo J, Liu K (2020). Hybrid radiofrequency ablation for a patient with WPW syndrome and complicated cardiovascular malformation. SN Comprehensive Clinical Medicine.

[8] Pappone C, Vicedomini G, Manguso F (2014). Wolff-Parkinson-White syndrome in the era of catheter ablation: insights from a registry study of 2169 patients. Circulation.

[9] Bass JE, Redwine MD, Kramer LA, Huynh PT, Harris JH Jr (2000). Spectrum of congenital anomalies of the inferior vena cava: cross-sectional imaging findings. Radiographics.

[10] Inama G, Vergara G, Gramegna L, Rillo M, Fuochi C, Furlanello F (1998). Catheter ablation of Wolff-Parkinson-White syndrome associated with congenital absence of inferior vena cava. J Interv Card Electrophysiol.

